# Brevundimonas diminuta-Induced Lung Abscess in an Immunocompetent Adult: A Rare Case Report

**DOI:** 10.7759/cureus.42371

**Published:** 2023-07-24

**Authors:** Mubariz A Hassan, Faisal Syed, Gagan P Singh, Ramya Pakala, Huda Gasmelseed

**Affiliations:** 1 Internal Medicine, Howard University Hospital, Washington, USA; 2 Internal Medicine/Gastroenterology, Howard University Hospital, Washington, USA

**Keywords:** immunocompetent adults, thoracic ct scan, brevundimonas diminuta, antibiotic treatment, • lung abscess

## Abstract

Lung abscesses caused by *Brevundimonas diminuta* (*B. diminuta*) are a rare occurrence, particularly in immunocompetent adults. We present the case of a 47-year-old male with a history of COPD, bipolar disorder, and seizure disorder, who presented with a productive cough, worsening shortness of breath, yellow sputum, weight loss, and fatigue over a period of three weeks. Clinical examination revealed decreased breath sounds in the left upper lung zones. Laboratory investigations showed an elevated white cell count, while blood cultures identified *B. diminuta*. Imaging with computed tomography (CT) confirmed the presence of a 4.2x2.0 cm cavitary lesion consistent with a lung abscess. The patient was successfully treated with a combination of Ampicillin/Sulbactam and Azithromycin, followed by a course of oral Augmentin. Given the size of the abscess and favorable response to antibiotic therapy, invasive procedures were deemed unnecessary. This case underscores the importance of considering unusual pathogens in the etiology of lung abscesses, even in immunocompetent individuals, and highlights the successful management with appropriate antibiotic therapy.

## Introduction

Lung abscesses are serious pulmonary infections characterized by the formation of a localized necrotic cavity within the lung parenchyma. They commonly occur secondary to aspiration, bronchial obstruction, or hematogenous spread of infection. While anaerobic bacteria, such as Streptococcus species and Bacteroides fragilis, are frequently implicated in lung abscesses, unusual pathogens can also be responsible, particularly in immunocompromised individuals.

Herein, we present a unique case of a lung abscess caused by *Brevundimonas diminuta* in an immunocompetent adult. *B. diminuta* is a Gram-negative bacillus found in various environmental sources, including water, soil, and hospital settings. Its association with lung abscesses is exceedingly rare and has been predominantly reported in immunocompromised hosts. The clinical presentation of our patient was notable for prolonged symptoms, including productive cough, weight loss, and fatigue, along with radiological evidence of a cavitary lesion on chest CT. Prompt diagnosis was achieved through blood cultures, which identified *B. diminuta* as the causative agent. We discuss the management approach, including antibiotic therapy, and the rationale for the absence of invasive procedures such as drainage intervention or bronchoscopy.

This case report highlights the need to consider uncommon pathogens, such as *B. diminuta*, in the etiology of lung abscesses, even in immunocompetent individuals. Increasing awareness among healthcare professionals can aid in accurate diagnosis and appropriate management of these rare cases, thereby optimizing patient outcomes.

## Case presentation

A 47-year-old male, bearing a significant medical history encompassing chronic obstructive pulmonary disease (COPD) managed intermittently with Albuterol inhalation therapy, as well as bipolar disorder and stable seizure disorder under the regimen of Depakote, presented with a protracted course of symptoms. These symptoms manifested as a productive cough, compounded by an exacerbation of dyspnea, persisting unabated for a duration exceeding three weeks. The patient, in his account, described the sputum to exhibit a disconcerting hue of yellow. Furthermore, exhaustive cataloging of his presenting complaints revealed an appreciable weight loss of 20 lbs (pounds) over the span of one month, alongside an overwhelming sense of fatigue.

The review of systems failed to uncover any concomitant symptoms of fever, chills, diarrhea, headaches, seizures, or any event suggestive of loss of consciousness. The physical examination revealed no signs of acute distress and evidenced vital signs within acceptable limits, encompassing a temperature of 98.4°F, heart rate of 112 beats per minute, respiratory rate of 18 breaths per minute, blood pressure measuring 110/67 mmHg, and arterial saturation at a commendable 99% while breathing ambient air. However, a notable finding emerged during auscultation of the pulmonary region, indicating diminished breath sounds localized to the left upper lung zones. On the other hand, evaluations encompassing the cardiac, abdominal, and neurological systems yielded unremarkable outcomes. Further workup with routine laboratory investigations including a comprehensive chemistry profile and complete blood count were diligently conducted. The patient was also tested negative for HIV infection. These investigations were significant for elevated white cell count, offering a notable clue (as demonstrated in Table 1).

**Table 1:** Laboratory results

Basic Labs	Results	Reference Range
White Blood Cells	14.41	3.2-10.6x10^9^/L
Hemoglobin	11.1	14.6-17.8 g/dL
Hematocrit	33.7	40.8-51.9%
Platelets	594	177-406x10^9^/L
Absolute Neutrophils	10.39	1.3-7.1x10^9^/L
Sodium	134	135-145 mEq/L
Chloride	99	95-111 mEq/L
Blood Urea Nitrogen	12	7-25 mg/dL
Creatinine	1.03	0.6-1.2 mg/dL

**Figure 1:**
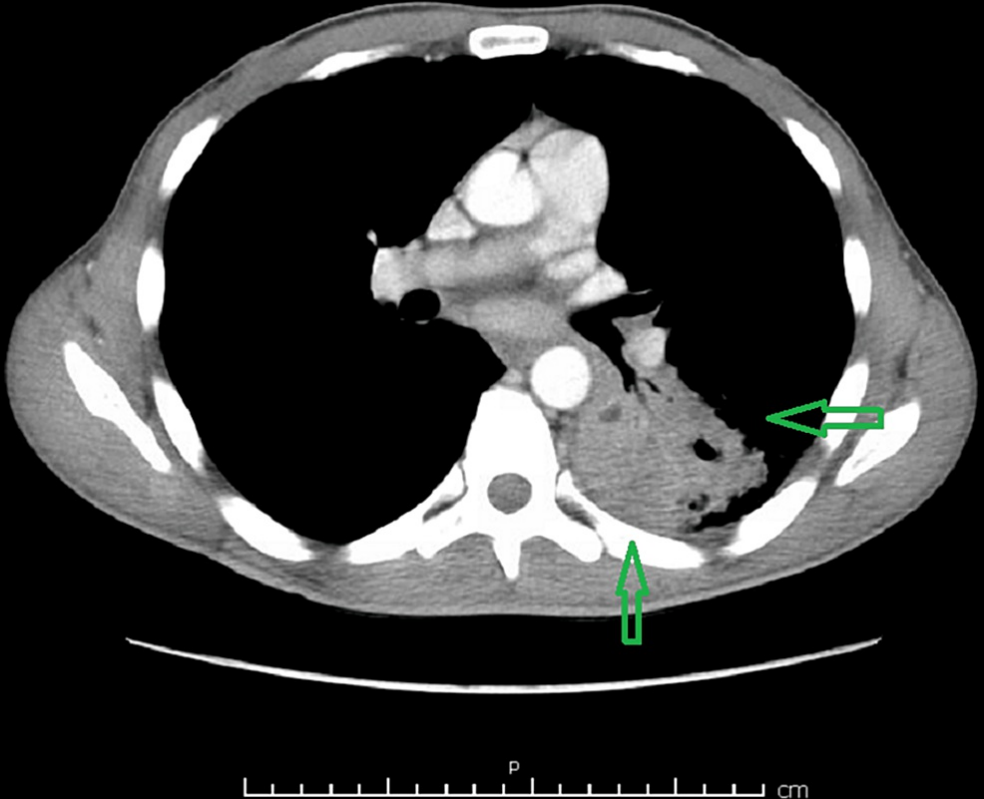
Computed tomography (CT) scan of the chest showing abscess cavity internal fluids or debris as shown with green arrows.

**Figure 2:**
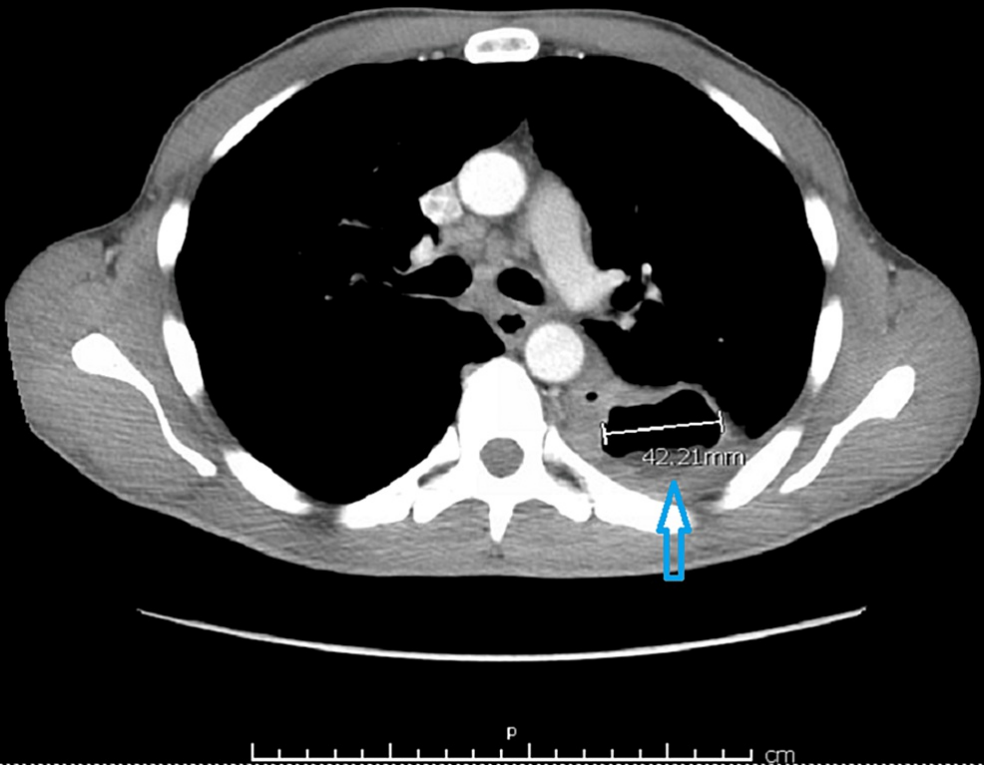
Computed tomography (CT) scan of the chest showing 4.2x2.0 cm interval cavitary process compatible with lung abscess as shown with blue arrow.

Furthermore, the pursuit of microbial identification via blood cultures proved consequential, as the cultures revealed the growth of *B. diminuta*, a discovery of paramount significance. Additionally, acid-fast bacillus (AFB) cultures, conducted in triplicate, were undertaken as part of the comprehensive workup but regrettably yielded negative results. Similarly, respiratory cultures yielded no aberrations, showcasing the growth of normal flora. To gain further insights into the morphological and structural underpinnings of the affliction, a computed tomography (CT) scan of the chest was conducted, revealing a dominant, discernible cavitary process measuring approximately 4.2 cm in its greatest dimension. Within this cavity, a discernible layer of internal fluid or debris was detected, thus corroborating the presence of a lung abscess (as illustrated in Figures 1, 2).

Considering the multifaceted nature of the patient's clinical presentation, a judicious treatment plan was formulated. During the patient's hospital stay, a therapeutic regimen comprising Ampicillin/Sulbactam and Azithromycin was administered initially. Upon discharge, the patient was instructed to complete a course of oral Augmentin spanning a duration of four weeks, aimed at ensuring a comprehensive resolution of the infectious process. Crucially, the patient underwent an exhaustive evaluation by the esteemed Pulmonology and Infectious Disease team, who concurred with the decision to continue the prescribed course of antibiotics, thereby eschewing any immediate drainage interventions or bronchoscopy. This decision was predicated upon the observed dimensions of the abscess, which were deemed amenable to pharmacological management, in conjunction with the favorable response to treatment, as ascertained through repeat imaging conducted subsequent to discharge. Consequently, the patient's convalescence was marked by prudent monitoring, with scheduled follow-ups within the purview of the esteemed Pulmonology team. Thus, the judicious implementation of targeted antibiotic therapy, promptly initiated in the early stages of management, remains a pivotal pillar for optimal patient outcomes in cases involving Brevundimonas-associated lung abscesses.

## Discussion

*B. diminuta*, belonging to the Brevundimonas genus, is a Gram-negative bacterium with distinctive straight, slim rod-shaped cells observed on gram staining. These organisms are aerobic, oxidase, and catalase positive, and do not form spores [1]. Another species within this genus is *Brevundimonas vescularis*, which has been implicated in supporting the growth of Legionella. On MacConkey agar, *B. diminuta* colonies exhibit a chalk-white appearance, while colonies of *B. vescularis* appear orange due to intracellular pigment. In clinical settings, the use of MALDI-TOF identification has proven effective in identifying Brevundimonas spp. [1].

*B. diminuta* primarily causes bacteremia; however, isolated cases of spontaneous bacterial peritonitis and cystic fibrosis have also been reported. It is an environmental organism commonly found in water, soil, plants, and occasionally in clinical specimens. Notably, it possesses the ability to survive in disinfectant solutions [2]. The intrinsic resistance of *B. diminuta* to quinolones has been documented in the literature, highlighting the importance of avoiding this class of antibiotics, particularly in immunocompromised patients [3].

In our presented case, the patient, an immunocompetent adult with a history of COPD, presented with a productive cough, worsening shortness of breath, yellow sputum, weight loss, and fatigue over a three-week period. Clinical examination revealed decreased breath sounds in the left upper lung zones. Laboratory investigations, including blood cultures, demonstrated an elevated white cell count, with *B. diminuta* identified as the causative agent. The CT scan confirmed the presence of a cavitary lesion consistent with a lung abscess. The treatment approach for our patient involved administering Ampicillin/Sulbactam along with Azithromycin to provide coverage for atypical organisms until ruled out. This therapeutic regimen led to marked clinical improvement in symptoms and a reduction in the size of the abscess. Subsequently, the patient was discharged with a four-week course of oral Augmentin and scheduled follow-up in the pulmonology clinic.

While *B. diminuta* is generally considered to have low virulence, as evidenced by the limited number of reported cases in recent times [4], it should not be overlooked as a potential causative agent of lung abscess in patients presenting with characteristic features. The successful management of our case, with resolution of symptoms and abscess reduction, highlights the importance of initiating targeted antibiotic therapy early in the course of treatment. It is noteworthy that lung abscesses caused by Brevundimonas species are relatively rare presentations [5]. However, raising awareness among healthcare professionals about the possibility of unusual pathogens, such as *B. diminuta*, can aid in the accurate diagnosis and appropriate management of these cases. Further research and clinical studies are warranted to better understand the pathogenesis, optimal treatment strategies, and outcomes associated with Brevundimonas-associated lung abscesses.

## Conclusions

*B. diminuta* is an infrequent cause of lung abscess, particularly in immunocompetent hosts. Prompt identification through septic workup and completion of a prolonged course of appropriate antibiotics remain crucial in the management of responsive patients with lung abscess. While invasive interventions and bronchoscopy may be necessary in some cases, our patient demonstrated favorable outcomes without the need for such procedures. Early initiation of targeted antibiotic therapy is essential in optimizing patient outcomes. Further research is warranted to delve deeper into the pathogenesis, optimal treatment strategies, and outcomes associated with lung abscesses caused by *B. diminuta*, thereby expanding our understanding of this rare clinical entity.
